# Infrared Devices Versus Traditional Palpation Approach for Peripheral Intravenous Catheter Insertion in Adults: A Systematic Review and Meta‐Analysis

**DOI:** 10.1111/jan.17007

**Published:** 2025-04-29

**Authors:** Bertrand Drugeon, Jessica A. Schults, Gillian Ray‐Barruel, Grace (Hui) Xu, Daner Ball, Hideto Yasuda, Rebecca Drugeon, Julie Mercier, Gabor Mihala, Natalie Barker, Olivier Mimoz, Claire M. Rickard

**Affiliations:** ^1^ CHU de Poitiers, Service des Urgences Adultes—SAS 86—Centre 15 Poitiers France; ^2^ INSERM, U1070, Pharmacologie des Agents Anti‐Infectieux Poitiers France; ^3^ Alliance for Vascular Access Teaching and Research Griffith University Nathan Queensland Australia; ^4^ School of Nursing, Midwifery and Social Work and Centre for Clinical Research University of Queensland Brisbane Queensland Australia; ^5^ Herston Infectious Diseases Institute, Metro North Health Herston Queensland Australia; ^6^ Nursing and Midwifery Research Centre, Royal Brisbane and Women's Hospital Herston Queensland Australia; ^7^ Emergency Trauma Centre Royal Brisbane and Women's Hospital Herston Queensland Australia; ^8^ Department of Emergency and Critical Care Medicine Jichi Medical University Saimata Medical Centre Saitama Japan; ^9^ Department of Clinical Research Education and Training Unit Keio University Hospital Clinical and Translational Research Centre (CTR) Tokyo Japan; ^10^ Centre for Health Services Research, Faculty of Medicine The University of Queensland Woolloongabba Queensland Australia; ^11^ Herston Health Sciences Library The University of Queensland Herston Queensland Australia

**Keywords:** clinical effectiveness, First‐attempt success, Infrared device, nursing assessment, Peripheral Intravenous Catheter (PIVC)

## Abstract

**Aims:**

This systematic review and meta‐analysis evaluated the efficacy of infrared (IR) devices versus the traditional palpation technique for first‐attempt success of peripheral intravenous catheter (PIVC) insertion in adults.

**Design:**

Systematic review and meta‐analysis of randomised controlled trials (RCTs).

**Data Sources:**

A comprehensive search of PubMed, Embase, Cochrane Library, Scopus and CINAHL was conducted on 28 May 2024 and included articles in English or French published from 1st January 2000 onwards.

**Review Methods:**

Eligible studies included RCTs comparing IR devices with the traditional palpation method for PIVC insertion in adults. The primary outcome was first‐attempt success. Secondary outcomes included overall success, number of attempts, cannulation time and patient pain. The risk of bias was assessed using the RoB2 tool, and a random‐effects model was applied for meta‐analysis.

**Results:**

Five RCTs were included, involving 690 patients and 704 catheters, including 289 PIVCs in patients with Difficult Intravascular Access (DIVA) criteria. First attempt insertion success was similar when using infrared devices (139/331, 42%) and traditional palpation (143/373, 38%) with Risk Ratio (RR) 1.08 (95% CI, 0.69 to 1.70). No significant statistical differences were noted in secondary outcomes: overall insertion success, number of attempts, time to cannulate and patient pain. Clinical and statistical heterogeneity were substantial (primary analysis *I* ^2^ = 83%).

**Conclusion:**

Current evidence does not support the systematic use of infrared devices to improve PIVC insertion success, reduce the number of attempts or alleviate patient pain compared with traditional palpation in adults. Further high‐quality studies with suitable sample sizes and varied populations are needed to better establish the potential place of infrared devices.

**Impact:**

This study highlights the limited benefit of IR devices in routine clinical practice and underscores the need for further research into their use in specialised settings.

**Patient or Public Contribution:**

No Patient or Public Involvement. This study did not include patient or public involvement in its design, conduct or reporting.

**Abbreviations**
CI: confidence intervals
DIVA: difficult intravascular access
IR: infrared
PIVC: peripheral intravenous catheters
PRISMA: preferred reporting items for systematic review and meta‐analysis
RCT: randomised controlled trials
RR: risk ratio
US: ultrasound
USA: United States of America

## 1 Introduction

Peripheral intravenous catheters (PIVC) are the most commonly used invasive medical devices in hospitals (Alexandrou et al. 2018). Every year, 2 billion catheters are sold worldwide (Alexandrou et al. 2015), with 60%–90% of hospitalised patients requiring peripheral vascular access (Helm et al. 2015). Although PIVC insertion is considered a routine healthcare procedure, it is often difficult, with 30% of first‐attempt insertions failing in adults and over 60% in children (Annalyn et al. 2024). Repeat insertion attempts are stressful and painful for patients and families, increase clinicians' workload, waste resources and increase the risk of complications, including nerve damage, arterial puncture, haematoma, thrombosis, scarring and catheter‐related infection (Helm et al. 2015; Cooke et al. 2018; van Loon et al. 2019; van Loon et al. 2018a; Schults et al. 2022). Risk factors for difficult intravascular access (DIVA) include chronic disease, patient factors (e.g., age extremes) and history of DIVA (Schults et al. 2022; van Loon et al. 2018b; Pitts and Ostroff 2019; Coulter 2016). Enablers for first attempt insertion success include access to equipment, skilled inserters and preassessment of risk.

Current practice for PIVC insertion is by landmark (‘blind’) technique, which uses palpation as the key step. Combined with visual inspection, palpation helps estimate the size, depth and accessibility of the vein. Ultrasound (US) is sometimes used when the initial assessment fails to identify a suitable vein for insertion, and should be routinely used in patients with DIVA criteria (van Loon et al. 2018b). The US allows the vein to be assessed prior to insertion and the needle to be visualised in real time during the procedure. US is associated with a higher success rate of the first cannulation attempt than palpation, particularly in DIVA patients, with less time required for successful catheter insertion and greater patient satisfaction (van Loon et al. 2018b; Paterson et al. 2022). Despite its promise and demonstrated efficacy in patients with DIVA, widespread adoption and implementation of US is slow due to inner and outer context factors such as the availability of professionals with US capability, as well as the availability and cost of equipment. Although compact and portable US equipment, ideal for PIVC insertion, is increasingly available, many institutions still rely on cumbersome machines mounted on heavy trolleys, which may limit their use in some patient locations, particularly for bedridden patients (Ray‐Barruel et al. 2024). In addition, additional organisational workflows are needed to maintain the hygiene and cleanliness of US devices, a task made more challenging with patients in contact isolation, especially those with COVID‐19 (Zhang et al. 2022).

Infrared (IR) devices, an alternate technology to support PIVC insertion, have been available for over a decade. These devices show a luminous glow on the patient's skin, tracing the path of veins. They are small and portable, can be mounted on a bracket to free up the operator's hands, and are easy to clean. IR devices have been mainly evaluated for pre‐anaesthetic intravenous cannulation of children (Annalyn et al. 2024; Park et al. 2016; Vyas et al. 2021); results were pooled in a meta‐analysis that showed first attempt insertion success, pain scores and duration of the procedure were similar between the IR devices and the palpation approach (Annalyn et al. 2024). To our knowledge, no review of the literature has been carried out in adults, and there is a need to clarify the value of using these devices for PIVC insertion in this population.

The aim of this systematic review and meta‐analysis was to compare the efficacy of IR devices with the landmark technique (traditional palpation) on first attempt insertion success, time to cannulate, number of attempts, pain during the procedure, and patient and caregiver satisfaction in hospitalised adults.

## 2 Methods

We performed a systematic review and meta‐analysis in full accordance with the Preferred Reporting Items for Systematic Review and Meta‐Analysis recommendations (PRISMA) (Page et al. 2021), further underpinned by Cochrane methods (Higgins et al. 2024). The protocol was registered in PROSPERO on June 1, 2024 (Ref: CRD42024549622).

### 2.1 Eligibility Criteria

We included randomised controlled trials (RCTs), including those with crossover design, comparing first attempt insertion success in adult patients (≥ 18 years or author‐defined) in hospital settings using an IR device or the traditional palpation approach. IR technology was defined as the use of a portable device to identify vein pathways and/or to guide the PIVC insertion procedure. Non‐randomised designs such as reviews, narrative reviews, meta‐analyses, observational studies, abstract‐only articles and conference abstracts were excluded. Studies on the insertion of central venous catheters, dialysis catheters or arterial catheters, and studies involving simulation models, animals or healthy volunteers were excluded. We anticipated that if five or more RCTs assessed first attempt insertion success, we would conduct a meta‐analysis.

### 2.2 Information Sources and Search Strategy

A systematic search was performed in the electronic databases PubMed, Embase, the Cochrane Library, Scopus and CINAHL using Medical Subject Headings (MeSH) and keywords related to vascular access and IR devices, such as “illuminator”, “viewer”, “infrared”, “near‐infrared”, “locating device”, “Catheterization, Peripheral”[Mesh], “PIVC” and “Catheter”[Mesh]. Database search strategies were developed with an information specialist (NB). The search was conducted on May 28, 2024, and included articles published from January 1, 2000, onwards, in English or French. The full details of the systematic search strategy for each database are provided in Supplementary File S1.

All database search results were imported into EndNote (Clarivate, Philadelphia), with a group created for the results from each database. This EndNote Library provides a complete record of the original database search results. An XML file was exported from EndNote for each group of database search results and imported into Covidence (Melbourne), which automatically removed duplicates prior to screening and displayed the number of results from each database source in the PRISMA flowchart.

### 2.3 Selection Process

Each title and abstract was independently assessed by two different review authors (BD, DB, GRB, JM, HY) based on the inclusion and exclusion criteria. In case of disagreement, a third reviewer (GRB, HY or BD), who was not part of the initial pair, was consulted to resolve discrepancies and reach a consensus. Next, the full text of selected articles was examined for eligibility by two review authors independently, based on the inclusion and exclusion criteria, with a third review author available to resolve discrepancies and achieve consensus.

### 2.4 Data Collection Process

Data from included articles were extracted by two independent review authors using a standardised data extraction tool in Covidence (BD and JM). Data extracted included study characteristics such as methods, country of publication, patient population, intervention and comparator descriptions, outcome definitions and statistical methods. When the results of data extraction differed between the first two review authors, a third review author checked the data in the articles (HY or GRB). Data were collated in Microsoft Excel (Microsoft, Washington) for statistical analysis.

### 2.5 Outcome Measures

The primary outcome was first attempt insertion success, defined as a functional PIVC inserted on the first attempt with no immediate complications (Zhang et al. 2022). Secondary outcomes were: (i) number of PIVC insertion attempts to achieve cannulation, (ii) overall insertion success, (iii) patient and inserter satisfaction of the PIVC insertion using a numerical scale, (iv) time to obtain a functional catheter defined as the time from the application of the tourniquet to the PIVC in place, and (v) patient pain during the procedure measured with a scale or tool, such as a Visual Analogue Scale.

Other collected data were study characteristics, including the name of the first author, date of publication, specialty (anaesthesia, emergency medicine, intensive care, conventional services), PIVC insertion risk (DIVA score), interventions and comparators used, and the number of patients included per arm. We reported the mean age of participants as age is a key factor influencing first‐attempt PIVC insertion success, with older adults often having fragile or sclerotic veins that increase insertion difficulty. Given that infrared devices are designed to enhance vein visibility, their effectiveness may vary depending on age‐related vascular characteristics, making this demographic detail essential for interpreting the results (Whalen et al. 2017).

### 2.6 Risk of Bias Assessment

Independently, two review authors or three if consensus was needed, assessed the risk of bias of all studies using the RoB2 tool for randomised studies, assessing potential bias due to the randomisation method, protocol deviations, missing data, measurement of the outcome and selection of the reported results (Sterne et al. 2019).

### 2.7 Statistical Analysis

#### 2.7.1 Raw Data Processing

Two or more studies were meta‐analysed for each research question. Continuous variables were presented as mean and standard deviation (SD). For studies where standard deviations were not reported, the SD was calculated from the Confidence Interval (CI) (Bland 2000). Categorical variables were described as numbers and percentages. Time to cannulate was converted into seconds when the results were in minutes. Pain scores used the published 0 to 10 pain scale (0 = no pain; 10 = the worst pain you have ever felt).

#### 2.7.2 Effect Measures

Measures of effect were assessed with a pooled Risk Ratio (RR) and 95% CI for dichotomous outcomes (success/failure). Weighted Mean Differences (WMD) and 95% CI were calculated for continuous data (cannulation time, number of attempts, pain and satisfaction). To select the most appropriate model, we first assessed clinical heterogeneity (defined below). Given that clinical heterogeneity was substantial, to perform the meta‐analysis, we chose a DerSimonian and Laird random‐effects model, although it overestimates the variance of the weighted average effect size when few studies are included in the meta‐analysis. Statistical heterogeneity was then investigated (Borenstein et al. 2010). A *p*‐value < 0.05 was considered statistically significant throughout the study. Forest plots were created to represent the results for each outcome, and tables were used to summarise characteristics of studies.

#### 2.7.3 Heterogeneity

Clinical heterogeneity was assessed by considering key factors that could influence study results: patient characteristics (age, DIVA criteria), clinical settings for PIVC insertions, and the training and experience of healthcare providers, which are crucial for success rates.

The statistical heterogeneity among studies was assessed using the *I* ^2^ statistic, where an *I* ^2^ value greater than 50% indicates significant heterogeneity (Higgins 2003). The standard errors of the study‐specific effect size estimates were adjusted to incorporate variation using the *τ* ^2^ (tau‐squared) variance (Borenstein et al. 2010; Higgins 2003). If statistical heterogeneity was present, we also considered sources of clinical or design heterogeneity and conducted a subgroup analysis on the primary outcome based on the following criteria:

– Size of studies: < 100 versus > 100 patients
– Population with DIVA patients versus population without DIVA patients
– Clinical setting of studies: Emergency department (ED) versus other wards

A sensitivity analysis was performed on the primary outcome to assess the robustness of the results. First, we excluded studies identified as having a high risk of bias according to the RoB2 tool, focusing on studies with issues related to randomisation, protocol deviations or incomplete outcome data. After excluding these studies, the meta‐analysis was repeated, and the results were compared to the original analysis. Second, we applied a fixed‐effects model to evaluate the consistency of the results compared to the random‐effects model used in the initial analysis. The fixed‐effects model assumes that the effect size is the same across all studies, which contrasts with the random‐effects model that accounts for variability between studies. Thirdly, we excluded the study with a crossover design.

#### 2.7.4 Software

Analyses were performed using R Studio (version 2024.04.2+764, Biocore, Boston) with the package *Metafor* (Viechtbauer 2010).

## 3 Results

### 3.1 Study Selection

**FIGURE 1:**
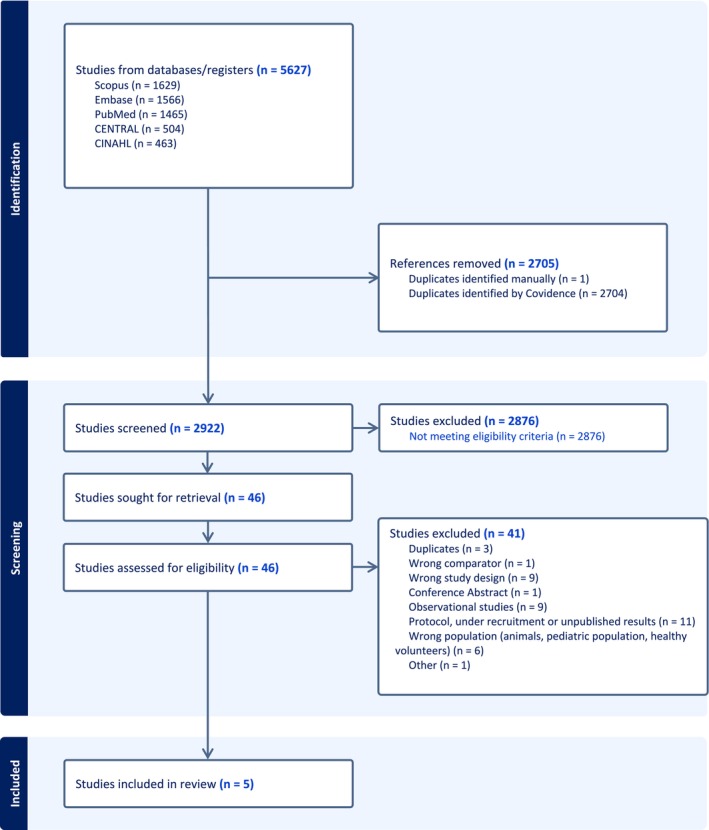
Flow chart of study identification and selection.

The search strategy resulted in 5627 studies. After eliminating 2705 duplicates and 2876 studies during title and abstract screening, 46 full‐text study articles were retrieved, with five meeting the eligibility criteria. Four review authors assessed French titles and abstracts for eligibility; however, no French‐language articles were selected for full‐text assessment or inclusion in the final analysis. Study flow is outlined in Figure 1 (flow chart). We found 11 studies in the process of being recruited or with unpublished results (the authors did not reply to emails asking for their results). The studies in progress could perhaps provide new perspectives on the use of these devices.

### 3.2 Study Characteristics

Characteristics of the five included studies are outlined in Table 1. In total, these studies included 690 patients and 704 PIVC insertions (289 in patients with DIVA), with 331 insertions using IR devices and 373 utilising the traditional approach. The average number of participants per study was 138. Two studies were conducted in French and Turkish emergency departments (ED) (Aulagnier et al. 2014; Yalçınlı et al. 2022), one in a surgical ward in the United States (USA) (Kanipe et al. 2018), one in an Indian medical ward (Rani et al. 2021), and one study in EDs, general wards and the Intensive Care Unit (ICU) in Iran (Al‐Saadi et al. 2022). All studies assessed the first attempt PIVC insertion and the time to cannulate; three studies assessed overall insertion success (Aulagnier et al. 2014; Rani et al. 2021; Al‐Saadi et al. 2022), three studies assessed the number of insertion attempts (Aulagnier et al. 2014; Yalçınlı et al. 2022; Al‐Saadi et al. 2022), two studies assessed patient pain (Aulagnier et al. 2014; Rani et al. 2021) and three studies assessed patient and staff satisfaction (Aulagnier et al. 2014; Kanipe et al. 2018; Rani et al. 2021). No patients from either the infrared device group or the traditional approach group were excluded from the analysis.

**TABLE 1:** Characteristics of included studies.

Authors	Year	Country	Clinical department	Study design	Comparators	Inserters		DIVA patients	Age^a^	Devices	Sample size	Primary outcome	Secondary outcomes
						Prof	Exp^a^						
Al Saadi and al.	2022	Iran	ED ICU General wards	RCT, 2 groups	Traditional approach Versus IR device	Nurses	NK	No	51	AccuVein	92	First attempt insertion success	Time to canulate Overall insertion success Number of attempts
Aulagnier and al.	2014	France	ED	RCT, 2 groups	Traditional approach Versus IR device	Nurses Students	7	Yes	57	Accuvein	272	Time to canulate	Number of attempts First attempt success Overall insertion success Patient's pain
Kanipe and al.	2018	USA	Surgery	RCT, 3 groups	Traditional approach Versus IR device Versus US	Nurses Doctors	NK	No	NK	AccuVein	90	First attempt insertion success	Time to canulate
Rani and al.	2021	India	General wards	Crossover RCT, 2 groups	Traditional approach Versus IR device	Nurses	NK	No	NK	Vein Viewer	86	Overall insertion success	Time to canulate Patient's pain First attempt insertion success
Yalcinli and al.	2022	Turkey	ED	RCT, 3 groups	Traditional approach Versus IR device Versus US	Nurses Doctors	7.8	Yes	66	AccuVein	270	First attempt insertion success	Time to canulate Number of attempts

Abbreviations: DIVA, difficult intravenous access; ED, emergency department; Exp, experience; ICU, intensive care unit; IR, infrared; Prof, profession; RCT, randomised controlled trial; US, ultrasound; USA, United States of America.

^a^ Data are means.

### 3.3 Risk of Bias in Studies

**FIGURE 2:**
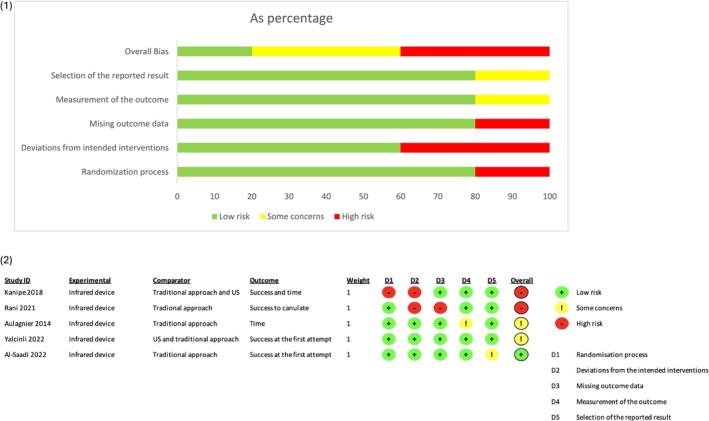
Risk of bias. Graph (1) and summary (2). Review the authors' judgment about each risk of bias item. US, ultrasound.

A Risk of Bias summary is provided in Figure 2. Items assessed as high risk of bias in one or more studies included the randomisation method, deviation from planned interventions and missing outcome data.

#### 3.3.1 Randomisation, Blinding and Concealment

Three studies declared doing randomisation with envelopes (Aulagnier et al. 2014; Yalçınlı et al. 2022; Kanipe et al. 2018), but only one reported using numbered opaque envelopes (Aulagnier et al. 2014). Two studies used a flipped coin to randomise (Rani et al. 2021; Al‐Saadi et al. 2022). In one study with a high risk of bias for randomisation, patients were not informed that they were in the study, and nurses could choose a method other than that allocated by randomisation (Kanipe et al. 2018). This same study also declared that if ‘the selected method was not used for any reason, the envelope was returned to the basket and the short peripheral catheter insertion attempt was not included in the data collection’ (Kanipe et al. 2018).

#### 3.3.2 Protocol Deviations and Missing Data

One study reported that 10 of 86 patients (12%) did not receive the allocated intervention, which is a considerable number of protocol deviations, and 26/86 patients (30%) were lost to follow‐up; therefore, 36/86 randomised patients (42%) could not be analysed. No intention‐to‐treat analysis was carried out (Rani et al. 2021).

#### 3.3.3 Measurement of the Outcome

First attempt insertion success was reported in all of the selected articles; however, definition heterogeneity was evident. Nevertheless, it is commonly accepted that this outcome is achieved when only one puncture is necessary to obtain a functional PIVC. Although the procedure is not blinded, this outcome is less prone to evaluator interpretation, and therefore cannot give an advantage to one group over another. However, only two studies used a clinical research nurse to measure outcomes (Aulagnier et al. 2014; Rani et al. 2021); in two other studies, this detail was not specified (Yalçınlı et al. 2022; Al‐Saadi et al. 2022); finally, in one study, the operator completed their own case report form (Kanipe et al. 2018), which can bias the results. The same applies to the number of attempts and overall insertion success. Time to PIVC insertion was defined as the time from tourniquet placement to a functional catheter in the five included studies, which is coherent and appropriate. This outcome was not assessed with blinding to the randomisation group, but it is sufficiently well defined to be considered objective, which may have had only a minimal impact on its measurement. Patient pain is a subjective concept, but using a numerical scale ranging from 0 to 10 is a common way of measuring pain (Aulagnier et al. 2014; Rani et al. 2021).

#### 3.3.4 Selection of the Reported Results

Only one study registered its protocol prior to the start of the study (Rani et al. 2021). The stated primary objective was twofold—to assess overall insertion success in both groups and patient satisfaction—but the statistical analysis was not detailed. These results are presented in the published article. The other studies did not publish a statistical analysis plan or register the primary endpoint definition in a clinical trials registry prior to patient recruitment. However, three studies reported results in accordance with their method section (Aulagnier et al. 2014; Yalçınlı et al. 2022; Kanipe et al. 2018). The statistical analysis section was unclear in one study; however, the results presented did not require any specific statistical analysis (Al‐Saadi et al. 2022).

### 3.4 Assessment of Reporting Biases

The evaluation of reporting biases was not carried out because this meta‐analysis includes fewer than 10 studies (Page et al. 2024).

### 3.5 Individual Outcomes and Synthesis

#### 3.5.1 Primary Outcome: First Attempt Insertion Success

All studies explored the first attempt insertion success, and this was the primary outcome in two studies (Yalçınlı et al. 2022; Al‐Saadi et al. 2022). One study included only patients with DIVA criteria (Yalçınlı et al. 2022), one study included 40% of DIVA patients (Aulagnier et al. 2014), while other studies included patients regardless of DIVA risk stratification (Kanipe et al. 2018; Rani et al. 2021; Al‐Saadi et al. 2022). In the IR devices group, 139/331 (42%) catheterisations were successful on first attempt compared with 143/373 (38%) in the traditional approach group, with RR 1.08 (95% CI, 0.69 to 1.70). Only one study showed results overwhelmingly in favour of IR devices (Al‐Saadi et al. 2022); however, the overall sample size was small (*n* = 92) and was not reported to be based on a power calculation. Heterogeneity between studies was substantial (*I* ^2^ = 83%). See Forest Plot 1.

**FOREST PLOT 1:**
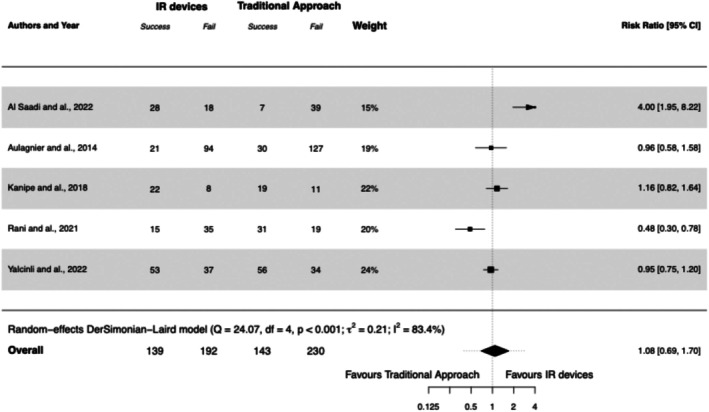
First attempt at insertion success using infrared (IR) devices compared to the traditional approach. IR, infrared.

A subgroup analysis for DIVA patients could not be conducted, as the studies involving this population did not report results specifically for these patients. Studies conducted in the ED or with a population over 100 patients are limited to those by Aulagnier et al. and Yalcinli et al., making the subgroup analyses based on these two factors identical. Analysis of this subgroup, encompassing a total of 452 patients, yielded a RR of 0.95 (95% CI, 0.77 to 1.17). These results are reported in the Supplementary File S2.

A sensitivity analysis excluding studies with a high risk of bias did not alter our results (RR 1.43 [95% CI, 0.70 to 2.92]). Similarly, excluding the crossover RCT had no impact on result interpretation (RR 1.30 [95% CI, 0.83 to 2.02]). Lastly, applying a fixed‐effect model also did not change the interpretation (RR 0.99 [95% CI, 0.84 to 1.16]). These findings are detailed in Supplementary File S3.

#### 3.5.2 Overall Insertion Success

Three studies explored overall insertion success (Aulagnier et al. 2014; Rani et al. 2021; Al‐Saadi et al. 2022) and this was the primary outcome in one study (Rani et al. 2021). Only one study reported participants' DIVA status, which represented more than half of the patients in this analysis (Aulagnier et al. 2014). Overall, there were 207/211 (98%) successful cannulations in the IR devices group compared with 247/253 (98%) in the traditional approach group. The use of IR devices was not associated with a higher rate of overall insertion cannulation success, with a RR of 1.00 (95% CI, 0.98 to 1.03), *I* ^2^ = 0%. See Forest Plot 2.

**FOREST PLOT 2:**
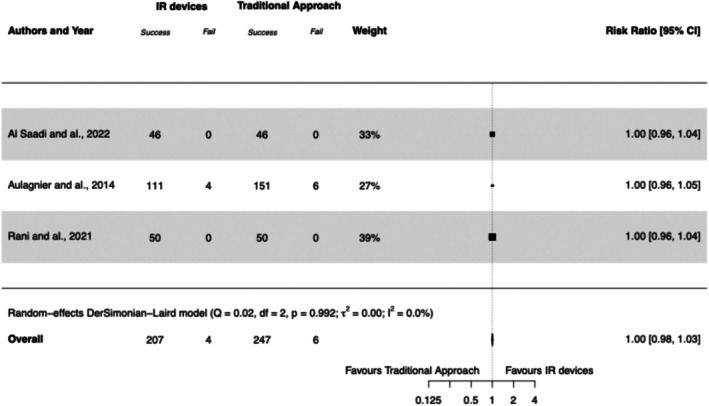
Overall insertion success of PIVC insertion using infrared (IR) devices compared to the traditional approach. IR, infrared; PIVC, peripheral intravenous catheter.

#### 3.5.3 Number of Attempts

Three studies reported the number of attempts needed for cannulation (Aulagnier et al. 2014; Yalçınlı et al. 2022; Al‐Saadi et al. 2022). One study included only patients with DIVA criteria (Yalçınlı et al. 2022), one study reported that 40% of patients met DIVA criteria (Aulagnier et al. 2014), and one study did not report the proportion of patients meeting DIVA criteria, but it included only obese patients with a BMI > 30 kg/m^2^ (Al‐Saadi et al. 2022). Overall, the mean number of attempts in the IR devices group was 1.3 compared with 1.6 in the traditional approach group. The use of IR devices was not associated with a reduction in the number of attempts compared with the traditional approach by palpation (WMD −0.34 [95% CI, −1.03 to 0.35], *I* ^2^ = 90%). See Forest Plot 3.

**FOREST PLOT 3:**
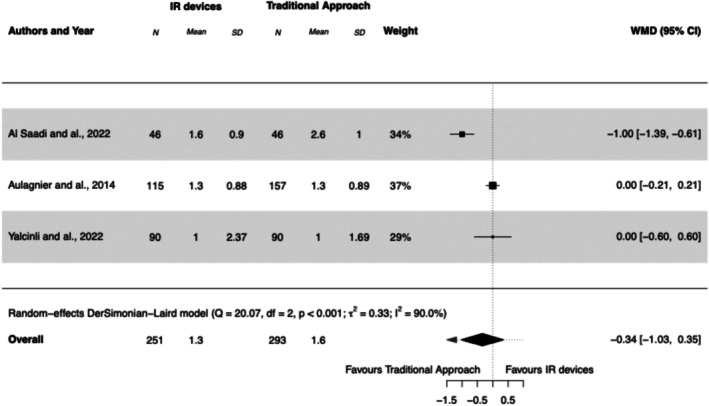
Mean number of attempts before successful PIVC insertion using infrared (IR) devices compared to the traditional approach. IR, infrared; PIVC, peripheral intravenous catheter; SD, standard deviation; WMD, weighted mean difference.

#### 3.5.4 Time to Cannulate

All studies calculated the time taken to cannulate, which was the main objective in one study (Aulagnier et al. 2014). One study reported a shorter time to cannulate in obese patients with IR devices (Al‐Saadi et al. 2022). Another study showed that the use of IR devices delayed the achievement of vascular access compared with the traditional approach by palpation in patients in a general ward, regardless of their venous status (Rani et al. 2021). Overall, the mean time to cannulate was 3.6 min in the IR devices group and 3.1 min in the traditional approach group. Use of IR devices was not associated with a reduction in cannulation time compared with the traditional approach (WMD 0.44 [95% CI, −0.58 to 01.47], *I* ^2^ = 94%). See Forest Plot 4.

**FOREST PLOT 4:**
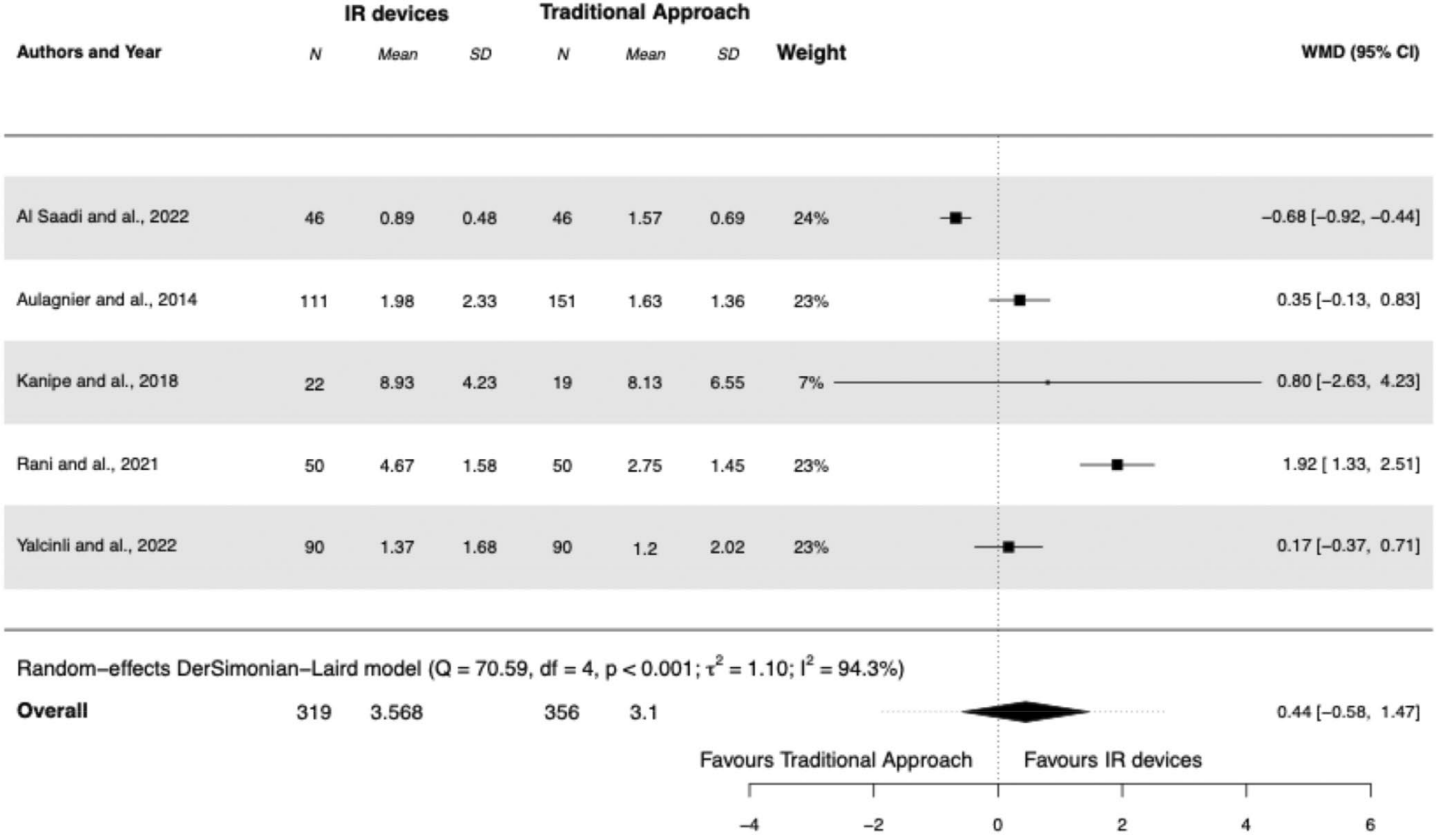
Mean time (in minutes) required to achieve successful PIVC insertion using infrared (IR) devices compared to the traditional approach. IR, infrared; PIVC, peripheral intravenous catheter; SD, standard deviation; WMD, weighted mean difference.

#### 3.5.5 Patient's Pain

Only two studies reported patients' pain (Aulagnier et al. 2014; Rani et al. 2021), and this was a secondary objective in both studies. The average pain experienced during PIVC insertion was 3.92 in the IR devices group and 3.49 in the traditional approach group. Overall, the use of IR devices tended to increase the pain experienced by patients (WMD 0.43 [95% CI, 0.03 to 0.83], *I* ^2^ = 0%). See Forest Plot 5.

**FOREST PLOT 5:**
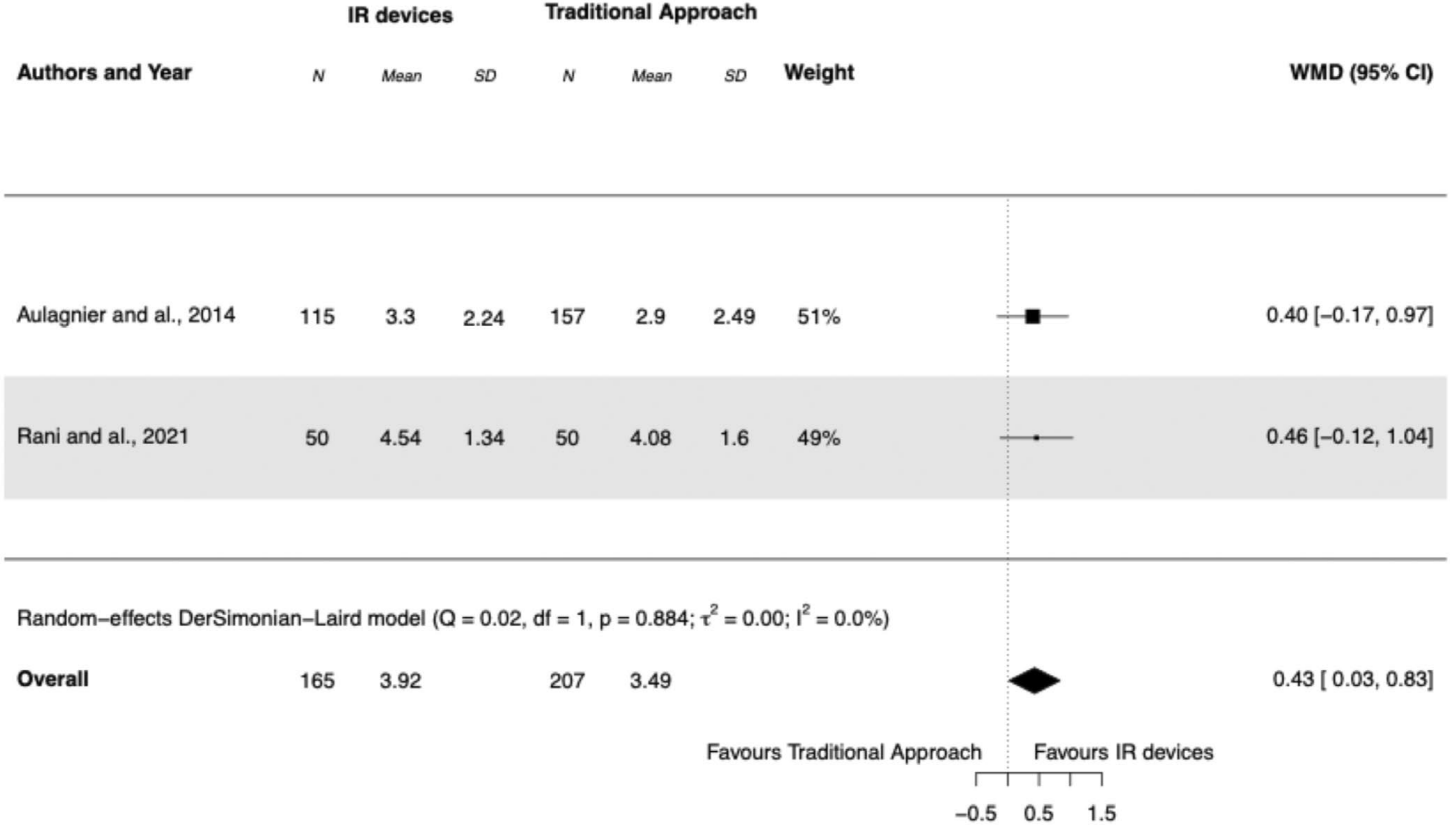
Mean pain score reported during PIVC insertion using infrared (IR) devices compared to the traditional approach. IR, infrared; PIVC, peripheral intravenous catheter; SD, standard deviation; WMD, weighted mean difference.

#### 3.5.6 Patient and Staff Satisfaction

Initially, we planned to analyse staff and patient satisfaction. However, of the three studies reporting this finding, the results were not comparable because the measurement method was too different, with different scales or ranges (Aulagnier et al. 2014; Kanipe et al. 2018; Rani et al. 2021). Rani et al. reported patient satisfaction scores on a 5‐point scale with an average of 3.28 in the IR devices group and 3.70 in the traditional approach group. Aulagnier et al. assessed nurses' satisfaction with IR devices using a questionnaire, without comparison with the control group; the overall assessment was negative or neutral among 73% of nurses. In 87% of cases, the use of the IR devices did not help in the choice of vein, and in 74% of cases, it did not help in visualising the vein. However, in 70% of cases it did not interfere with vein visualisation and in 68% of cases it did not interfere with puncture. Kanipe et al. allowed a free‐text assessment of the inserters.

## 4 Discussion

This is the first systematic review and meta‐analysis to evaluate the efficacy of IR devices for PIVC insertion in adults compared to traditional vein palpation. The review included five RCTs involving 704 catheters in 690 patients (289 were classified as DIVA patients). IR devices did not increase the first attempt insertion success, with rates of 42% for IR devices and 38% for the traditional palpation approach. Similarly, IR devices did not improve overall insertion success, reduce cannulation time or reduce patient pain during insertion. Consequently, current evidence does not support the routine use of IR devices, although most studies were small (< 100 patients), lacked clear differentiation between DIVA and non‐DIVA patients or between novice and experienced inserters, and two studies had a high risk of bias. These findings are particularly relevant for nursing practice, as PIVC insertion is a common nursing procedure performed worldwide. By providing evidence that IR devices do not enhance insertion success, this review supports the need to prioritise other strategies, such as specialised training in traditional techniques and US guidance, to improve PIVC outcomes in clinical practice.

### 4.1 Primary Outcome

All five studies reported first attempt insertion success; however, only one reported higher success with an IR device than with the traditional method (Al‐Saadi et al. 2022). Overall, the use of IR devices did not significantly improve first attempt insertion success (RR 1.08 [95% CI, 0.69 to 1.70]). This finding remained consistent across subgroup analyses by ED settings, studies with over 100 patients, and DIVA patients (Aulagnier et al. 2014; Yalçınlı et al. 2022), as well as in sensitivity analyses. Likewise, a recent meta‐analysis in paediatric patients, comprising 18 RCTs and nearly 5300 participants, found that IR devices did not significantly enhance first attempt insertion success (RR 1.05 [95% CI, 0.97 to 1.14]) (Annalyn et al. 2024).

Conversely, Al Saadi et al. reported favourable outcomes for IR devices, contrasting with the broader findings of this review. They observed a first attempt insertion success rate of 61% with IR devices versus 15% with the traditional approach in obese and diabetic patients, although their study lacked details on sample size and recruitment. Additionally, an observational study with a similar objective reported significant results, with a success rate of 77% in the control group and 92% in the IR devices group (Zhang et al. 2022). However, this study focused on isolated patients with severe COVID‐19, excluding DIVA patients. The use of personal protective equipment, such as double gloves, fogged goggles and corrective glasses restrictions, may impact tactile and visual precision, making IR devices advantageous due to their portability and ease of cleaning compared with traditional cart‐mounted US machines (Drozd et al. 2021).

Expertise in vascular access is known to influence insertion success (Jacobson and Winslow 2005), IR devices might benefit settings with less experienced inserters, such as students or recent graduates, potentially reducing attempts and improving success rates. However, no RCTs have yet evaluated IR devices among novice inserters. Aulagnier et al. and Yalcinli et al. noted that inserters in their studies averaged 7 years of experience, suggesting skilled professionals (Aulagnier et al. 2014; Yalçınlı et al. 2022). In Aulagnier et al., nurses performed approximately 27 catheter insertions weekly and rated their competence at 7/10. However, students constituted 17% of the control group and 10% of the intervention group, which might have affected outcomes, particularly cannulation time (Aulagnier et al. 2014).

Conceivably, IR devices might specifically benefit DIVA patients, yet few studies reported DIVA criteria, limiting assessment of a potential effect in this high‐risk group. A single factor, such as obesity (Al‐Saadi et al. 2022) is insufficient to define DIVA status (van Loon et al. 2019). Yalcinli et al. included 87% of patients with two or more DIVA criteria from previous research (Yalçınlı et al. 2022), while Aulagnier et al. included 43% of patients with prior puncture difficulties and 54% with low vein visibility (Aulagnier et al. 2014). In contrast, Kanipe et al. and Rani et al. did not report DIVA patient proportions in their cohorts (Kanipe et al. 2018; Rani et al. 2021). The likelihood of first attempt insertion success decreases as DIVA criteria increase and improves with US use (Paterson et al. 2022; van Loon et al. 2016). Systematically identifying DIVA patients with a validated tool is essential to optimise insertion success from the outset. Although DIVA patients often lack visible veins and could potentially benefit from IR devices, the studies did not perform subgroup analyses to assess the impact of IR devices specifically in DIVA patients. Furthermore, as IR devices highlight only superficial veins, which are limited in DIVA patients, their effectiveness may be constrained.

Our findings of the limited effect of IR devices on first attempt insertion success contrast sharply with US guidance, which has been consistently supported by multiple systematic reviews. A recent systematic review and meta‐analysis, including seven studies and nearly 1000 patients, showed that US guidance significantly improved first attempt insertion success (OR 3.07 [95% CI, 1.66 to 5.65]) (Poulsen et al. 2023). Another review of 20 studies, mostly from the USA, reported a 72% first attempt success rate in 19 studies; a meta‐analysis of two of these studies (300 patients) demonstrated an OR 0.42 (95% CI, 0.25 to 0.70), favouring US over the traditional approach (Álvarez‐Morales et al. 2024). In our review, two studies compared traditional methods, IR and US; Kanipe et al. reported 73% success with IR and 67% with US; the limited duration of US training, typically 1 h, may have impacted outcomes, as US generally requires 15–26 insertions for proficiency (Stolz et al. 2015). Yalcinli et al. similarly reported a 79% success rate with US and 59% with IR among DIVA patients. While IR devices are simpler, quicker to use and require minimal training or skin contact, their application remains limited to superficial veins, reducing suitability for high‐risk cases. In critical care, where deeper imaging is essential, US is preferred for its ability to visualise deeper veins. IR devices, while affordable and easy to deploy (Cantor‐Peled and Ovadia‐Blechman 2016), are more practical in resource‐limited or pre‐hospital settings or as a complement to US in straightforward cases. Further research should explore these specific applications, with US continuing to be recommended for DIVA patients, especially when performed by experienced professionals.

### 4.2 Secondary Outcomes

IR devices did not significantly improve overall insertion success compared to the traditional palpation approach (RR 1.00 [95% CI, 0.98 to 1.03]), although comparisons are complicated by varying definitions of success across studies. In some, success was defined by puncture count, such as fewer than five or fewer than three attempts (Aulagnier et al. 2014; Al‐Saadi et al. 2022), or by cannulation time, but these criteria were not consistently specified, and protocol deviations, such as patient exclusions, further influenced results. Some studies also restricted attempts to hand veins, potentially impacting outcomes (Rani et al. 2021). Notably, stricter definitions tend to increase failure rates, especially with traditional methods, while in some cases, rescue procedures were more frequent in the IR group, suggesting different approaches to handling failed attempts (Yalcinli). These findings highlight the need for a standardised definition of PIVC insertion success to improve comparability and support decision‐making.

Furthermore, as overall insertion success is frequently achieved regardless of technique, it may serve as an inadequate evaluation metric, failing to capture the complexities and resource demands involved. A focus beyond success rates alone might better reflect the challenges faced, such as material waste and operator time, as repeated attempts and initial failures can increase resource use and disrupt workflows. Repeated PIVC insertion failures or extended IR device use can also add strain on healthcare personnel, especially in high‐stress settings like ICUs or busy EDs. Therefore, evaluations of IR devices should also address their potential to reduce staff stress and workload alongside patient benefits. Future studies should consider broader criteria beyond first‐attempt insertion success, incorporating measures of clinical efficiency, resource optimisation (minimising waste and time), and impacts on healthcare organisations. Such a holistic approach could provide a clearer understanding of the value of IR devices, taking into account technical, economic and human factors within clinical practice.

The number of PIVC insertion attempts was not significantly impacted by IR devices compared to the traditional approach (WMD −0.34 [95% CI, −1.03 to 0.35]), with clinical practice generally aiming for a single attempt. Al Saadi et al. reported higher first‐attempt insertion success with IR devices, with an average of 1.6 attempts in the IR group versus 2.6 with the traditional approach. However, in Aulagnier and Yalcinli's studies, attempts averaged between 1 and 1.3 across groups, suggesting less variation. This differs from findings in a paediatric meta‐analysis, where IR use reduced the number of attempts by −0.47 (95% CI, −0.79 to −0.14) (Annalyn et al. 2024), likely because paediatric patients present additional challenges, such as smaller veins and lower cooperation, which can amplify the benefits of IR devices. In adults, with generally more visible veins, the advantage of IR devices is less pronounced. Additionally, training and experience appear to influence outcomes: in Aulagnier and Yalcinli's studies, nurses received specific training with IR devices, likely improving efficiency, while training details were not provided in Al Saadi's study.

Minimising the number of attempts is essential, as fewer attempts reduce patient discomfort, material waste and procedural time, thereby lowering the risk of infection, haematomas and thrombosis (van Loon et al. 2016). Although success is eventually achieved, reducing attempts is key to optimising outcomes. Paediatric data suggest IR devices may be particularly beneficial in cases requiring enhanced vein visualisation, such as with young patients, whereas their advantages in adults, especially among experienced operators, appear more limited. Further research should evaluate IR device effectiveness across different age groups to determine optimal, resource‐efficient applications.

IR devices did not significantly affect cannulation time compared to the traditional approach (WMD 0.44, [95% CI, −0.58 to 1.47]), even among DIVA patients (Aulagnier et al. 2014; Yalçınlı et al. 2022). In contrast, a recent paediatric meta‐analysis showed IR devices reduced cannulation time (29.4 s (0.49 min), [95% CI, −57.5 to −1.3]), although this difference is clinically negligible and likely due to anatomical differences between adults and children (Annalyn et al. 2024). Differences in time measurement methods also influenced results; for instance, Al Saadi et al. measured time to needle insertion, while others measured until functional catheter insertion, resulting in shorter times in Al Saadi's study that favoured IR devices. Overall, reported cannulation times were short, likely because they did not account for time spent on failed attempts, which would provide a more accurate reflection of the actual time impact. Some studies found higher cannulation times and failure rates with IR devices; Rani et al. reported that 70% of IR group patients required two or more attempts, compared to 38% in the traditional group. Similarly, US increased cannulation time by 30 s to 3 min in Yalcinli and Kanipe's studies due to additional setup and hygiene requirements.

Establishing a clinically significant threshold for cannulation time differences remains challenging and context‐dependent. A 30‐s delay might seem minor in routine care, yet even small time savings can be crucial in high‐pressure environments like ICUs or EDs. Measuring cannulation time from the initial request for access, rather than from tourniquet placement, might also provide a broader perspective on delays, especially for DIVA patients, where resource availability can add critical time in urgent settings. This expanded measurement approach could better capture the potential efficiency benefits of IR devices.

IR devices did not significantly impact pain levels during catheter insertion (WMD 0.43, [95% CI, 0.03 to 0.83]), as observed in two studies using the same pain assessment method (Aulagnier et al. 2014; Rani et al. 2021). Although the difference in pain was statistically significant, its clinical relevance appears limited. The correlation between first‐attempt insertion success and lower pain is well‐established: patients who experience successful first‐attempt insertions report less pain, whereas DIVA patients or those requiring multiple attempts typically report higher discomfort (Fields et al. 2014). Stratified pain reporting that emphasises those experiencing greater discomfort could provide insights beyond average pain scores. Future studies should consider more comprehensive pain scales that capture cumulative pain from repeated attempts, rather than focusing solely on single‐insertion pain, which would better guide clinical decisions on using IR devices for specific patient groups.

This review was unable to compare patient and staff satisfaction due to varying measurement methods across studies. However, available data indicate that nurses often rated IR devices negatively, frequently citing their perceived ineffectiveness. This sentiment may partly reflect a confidence in traditional insertion techniques. Notably, no studies to date have explored the use of IR devices solely for pre‐assessment rather than insertion, particularly with novice or less frequent PIVC inserters in clinical practice. The work environment likely influences satisfaction levels as well. In high‐pressure settings such as EDs, IR devices may seem redundant if they do not demonstrably improve insertion efficiency; conversely, in less demanding environments or among less experienced staff, IR devices might offer valuable visual guidance. Negative evaluations from nurses could also stem from unmet expectations: if nurses anticipated significant benefits, such as fewer insertion attempts or higher success rates, but observed minimal impact, dissatisfaction may result, even if IR devices offer certain advantages in specific situations.

### 4.3 International Guidelines on Use of Technology for PIVC Insertion

International guidelines, particularly from the Infusion Nurses Society (INS), endorse the use of specific technologies to improve PIVC insertion success and reduce complications, especially for DIVA patients (Nickel et al. 2024). US is recommended as the preferred technology for high‐risk cases due to its effectiveness in enhancing first‐attempt insertion success and its ability to visualise deeper veins and surrounding structures, such as arteries and nerves. INS guidelines suggest using US to assess vein size, calibre and patency prior to insertion, helping clinicians select the optimal vessel and guide insertion with minimal tissue damage.

Visible light devices may aid in locating superficial veins, particularly in neonates, though their utility decreases with age due to thicker tissue. Infrared devices can support DIVA cases by revealing vein details like bifurcations and valves; however, current evidence does not yet justify their routine use over traditional methods or US. Thus, US remains the technology of choice according to international standards, while further research is needed to evaluate the potential of near‐infrared in specific settings.

To optimise the integration of adjunctive technologies such as IR devices and US in clinical practice, the development of a structured PIVC insertion checklist could guide device selection and procedural decision‐making. Although beyond the scope of this study, future research should aim to define and validate such a tool. This checklist could incorporate key elements such as patient vascular access difficulty (DIVA criteria), operator expertise, device availability, urgency of the procedure and prior failed attempts. By ensuring systematic assessment of these parameters, this approach could help reduce variability in clinical decision‐making, promote appropriate technology use and improve patient outcomes.

### 4.4 Limitations of the Evidence and Heterogeneity

The overall quality of the evidence was judged to be low due to the small number of studies and significant bias in some trials. Our confidence in the findings is also limited by wide confidence intervals and high heterogeneity. The intervention's nature prevented blinding, which may have influenced results. However, we focused on first‐attempt insertion success, a clear and objective outcome.

Heterogeneity was present in several areas. Sample sizes varied, with only two studies including over 100 patients, accounting for two‐thirds of the total participants, and they were the least biased. Some studies compared three groups (traditional approach, IR devices and US guidance), affecting statistical power if not calculated based on the relevant groups. Additionally, the studies took place in different wards (surgical, ICU, ED), leading to variability in patient characteristics. In emergency settings, patient flow pressure may have complicated recruitment. Nurse experience also varied, with only two studies reporting it; one included students and none involved doctors. Lastly, training in the use of IR devices was inconsistent, ranging from brief sessions to 4‐h courses with validation, and no study included reminders on traditional PIVC practices, potentially affecting the results.

Given the heterogeneity observed in the included studies, establishing a structured minimum data set for PIVC‐related research would help standardise key variables, enhance comparability across studies and support the development of predictive models for PIVC insertion outcomes. Standardising data collection across studies could also improve evidence synthesis and inform future research on the most effective strategies for optimising PIVC insertion practices in different clinical settings.

### 4.5 Limitations of the Review Process Used

This study presents several important methodological limitations. Firstly, the restriction to studies published only in French and English may have led to the exclusion of relevant research in other languages, thereby limiting the scope of the conclusions. Secondly, significant heterogeneity in study designs was observed, with variations in the definition of outcomes, such as cannulation time and success rates, making direct comparisons between studies difficult and weakening the robustness of the conclusions. However, we performed an analysis using a DerSimonian and Laird random‐effects model to account for this heterogeneity. Furthermore, when heterogeneity was too high, we conducted subgroup analyses. Thirdly, the statistical power was also limited, particularly for outcomes such as the number of attempts and pain, where only a few studies were available, reducing the ability to detect meaningful differences. Finally, the review primarily focused on RCTs, excluding high‐quality observational studies or more innovative research, which may have restricted the diversity of perspectives and results obtained.

### 4.6 Strengths of the Study

Our study has several strengths. First, the rigorous article search process, conducted by an information specialist, ensured an exhaustive and high‐quality selection of included studies. Second, the use of IR devices is standardised in the selected studies, with 4 out of the 5 studies using the same brand device (AccuVein, Medford), which strengthens the consistency of the results. Third, the study evaluated several relevant clinical outcomes, such as cannulation time, first attempt insertion success and patient pain. This multidimensional approach provides a comprehensive view of the impact of IR devices on PIVC insertion. Fourth, the use of robust quantitative methods, such as the random‐effects model by DerSimonian and Laird, effectively addresses the heterogeneity among studies, ensuring more reliable estimates. Fifth, the study explores specific subgroups, such as patients with DIVA and wards, highlighting the potential relevance of IR devices in these particular clinical contexts. Finally, by acknowledging the limitations of the current data, such as the limited impact on pain and the contradictory findings regarding cannulation time, the study opens up avenues for future research aimed at improving care for patients with complex venous access needs.

## 5 Conclusion

This systematic review and meta‐analysis indicate that IR devices do not outperform the traditional approach for PIVC insertion in adults. Currently, IR devices should not be part of the standard venous access approach, particularly given that US remains the preferred method, especially for DIVA patients. While this review does not prompt immediate clinical or policy changes, it highlights the need for more research to assess IR devices' potential role. Future high‐quality studies should examine whether IR devices benefit specific patient or operator subgroups and compare IR directly with US, the gold standard, in challenging cases like DIVA. Until stronger evidence is available, IR devices should be used cautiously, not as replacements for established techniques like US.

## Author Contributions

All authors substantially contributed to conception and design, or acquisition of data, or analysis and interpretation of data; and gave final approval of the version to be published. Each author should have participated sufficiently in the work to take public responsibility for appropriate portions of the content. B.D. agreed to be accountable for all aspects of the work to ensure that questions related to the accuracy or integrity of any part of the work are appropriately investigated and resolved. B.D., J.A.S., G.M., O.M. and C.M.R. involved in drafting the manuscript or revising it critically for important intellectual content.

## Disclosure

Registration and protocol: The protocol can be consulted on PROSPERO, where it was registered on 01/06/2024 with reference CRD42024549622. We had initially planned to also select observational studies, but we decided to exclude them during the selection process. We have recorded this change in PROSPERO.

## Conflicts of Interest

C.M.R.'s employer (The University of Queensland or Griffith University) has received unrestricted research grants on her behalf from Solventum (formerly 3M), BD, Cardinal Health, Eloquest; consultancy payments from 3M, BD, BBraun and ITL Biomedical; education grants for the AVATAR Group from Angiodynamics, Solventum, ICU Medical and Spectrum Vascular; and product donations from Christie Medical. O.M. and B.D. received funding for congress attendance and research funding from Becton Dickinson and 3M. D.B. reports prior consultancy payments provided to her employer (The University of Queensland) from product manufacturers Solventum (formerly 3M), BBraun, Becton Dickinson and Terumo. G.R.‐B. reports prior consultancy payments provided to her employer (The University of Queensland or Griffith University) by product manufacturers (Solventum [formerly 3M], Becton Dickinson, BBraun) and education providers (Ausmed, Wolters Kluwer). J.M., J.A.S., R.D., D.B., G.H.X., G.M., N.B. and H.Y. have no conflicts of interest.

## Supporting information


**File S1.** Research strategy.

**File S2.** Subgroup analysis for first attempt insertion success using infrared (IR) devices compared to traditional approach in (1) emergency departments (EDs) and (2) other wards.

**File S3.** Sensitive analysis for first attempt insertion success using infrared (IR) devices compared to traditional approach: (1) without studies with high‐risk bias; (2) without the study with crossover design; (3) with fixed effect model analysis.

## Data Availability

The datasets generated and analysed during the current study, including template data collection forms, data extracted from the included studies, data used for all analyses, analytic code and any other materials used in the review, are not publicly available but can be provided by the corresponding author upon reasonable request. Researchers interested in accessing these materials for further analysis or validation purposes are encouraged to contact the corresponding author directly.
