# Supplementary material for: Infrared Devices Versus Traditional Palpation Approach for Peripheral Intravenous Catheter Insertion in Adults: A Systematic Review and Meta‐Analysis
Source: J Adv Nurs. 2025 Apr 29;82(2):1775–90. doi: 10.1111/jan.17007 (PMC12810616; doi:10.1111/jan.17007)
Supplement: Supplementary file 1 — File S1. Research strategy. [file JAN-82-1775-s002.docx]

Supplementary file 1. Research strategy.

**Databases**

PubMed, Embase, Cochrane Library, CINAHL Complete and Scopus

**Date searched**

28 May 2024

**Total number of results from database searches = 5627**

Imported into Covidence, which removed duplicates.

**Number of results after duplicates removed = 2923**

**PubMed** 1465 results

Includes MeSH

(("Veins"[Mesh] OR “vein”[tiab] OR “veins”[tiab] OR “venous”[tiab] OR “vascular”[tiab] OR “intravenous”[tiab]) AND ("Infrared Rays"[Mesh] OR “illuminator”[tiab] OR “illuminators”[tiab] OR “viewer”[tiab] OR “viewers”[tiab] OR “viewing”[tiab] OR “visualiser”[tiab] OR “visualisers”[tiab] OR “visualizer”[tiab] OR “visualizers”[tiab] OR “visualisation”[tiab] OR “visualization”[tiab] OR “finder”[tiab] OR “locating device”[tiab] OR “locating devices”[tiab] OR “infrared”[tiab] OR “near-infrared”[tiab] OR “near infrared”[tiab] OR “light”[tiab] OR “heat wave”[tiab] OR “heat waves”[tiab] OR “AccuVein”[tiab]) AND ("Catheterization"[Mesh] OR "Catheterization, Peripheral"[Mesh] OR "Catheters"[Mesh] OR "Vascular Access Devices"[Mesh] OR “cannulation”[tiab] OR “cannulations”[tiab] OR “catheter”[tiab] OR “catheters”[tiab] OR “catheterisation”[tiab] OR “catheterization”[tiab] OR “vascular access”[tiab] OR “venous access”[tiab] OR “PIVC”[tiab] OR “PIVCS”[tiab] OR “PVC”[tiab] OR “PVCS”[tiab]) AND (eng[la] OR fre[la] OR und[la]) NOT (animals [mh] NOT humans [mh]) AND 2000:2024[dp]) NOT (“child”[ti] OR “children”[ti] OR “childhood”[ti] OR “pediatric”[ti] OR “paediatric”[ti] OR “pediatrics”[ti] OR “paediatrics”[ti] OR “neonatal”[ti] OR “neonate”[ti] OR “neonates”[ti] OR “newborn”[ti] OR “newborns” OR “infant”[ti] OR “infants”[ti] OR “juvenile”[ti])

**Embase (Elsevier)** 1566

Includes Emtree

Limited to relevant publication types for full papers (including articles, articles in press and reviews).

(('vein'/exp OR “vein”:ti,ab OR “veins”:ti,ab OR “venous”:ti,ab OR “vascular”:ti,ab OR “intravenous”:ti,ab) AND ('infrared radiation'/exp OR “illuminator”:ti,ab OR “illuminators”:ti,ab OR “viewer”:ti,ab OR “viewers”:ti,ab OR “viewing”:ti,ab OR “visualiser”:ti,ab OR “visualisers”:ti,ab OR “visualizer”:ti,ab OR “visualizers”:ti,ab OR “visualisation”:ti,ab OR “visualization”:ti,ab OR “finder”:ti,ab OR “locating device”:ti,ab OR “locating devices”:ti,ab OR “infrared”:ti,ab OR “near-infrared”:ti,ab OR “near infrared”:ti,ab OR “light”:ti,ab OR “heat wave”:ti,ab OR “heat waves”:ti,ab OR “AccuVein”:ti,ab) AND ('catheterization'/exp OR 'catheter'/exp OR 'vascular access device'/exp OR “cannulation”:ti,ab OR “cannulations”:ti,ab OR “catheter”:ti,ab OR “catheters”:ti,ab OR “catheterisation”:ti,ab OR “catheterization”:ti,ab OR “vascular access”:ti,ab OR “venous access”:ti,ab OR “PIVC”:ti,ab OR “PIVCS”:ti,ab OR “PVC”:ti,ab OR “PVCS”:ti,ab) AND ([english]/lim OR [french]/lim) NOT (‘animal experiment’/de NOT (‘human experiment’/de OR ‘human’/de)) AND [2000-2024]/py) NOT (“child”:ti OR “children”:ti OR “childhood”:ti OR “pediatric”:ti OR “paediatric”:ti OR “pediatrics”:ti OR “paediatrics”:ti OR “neonatal”:ti OR “neonate”:ti OR “neonates”:ti OR “newborn”:ti OR “newborns” OR “infant”:ti OR “infants”:ti OR “juvenile”:ti) AND ([article]/lim OR [article in press]/lim OR [review]/lim)

**Cochrane Library (Wiley) CENTRAL** 504 Trials

Includes MeSH

Advanced search > Search manager


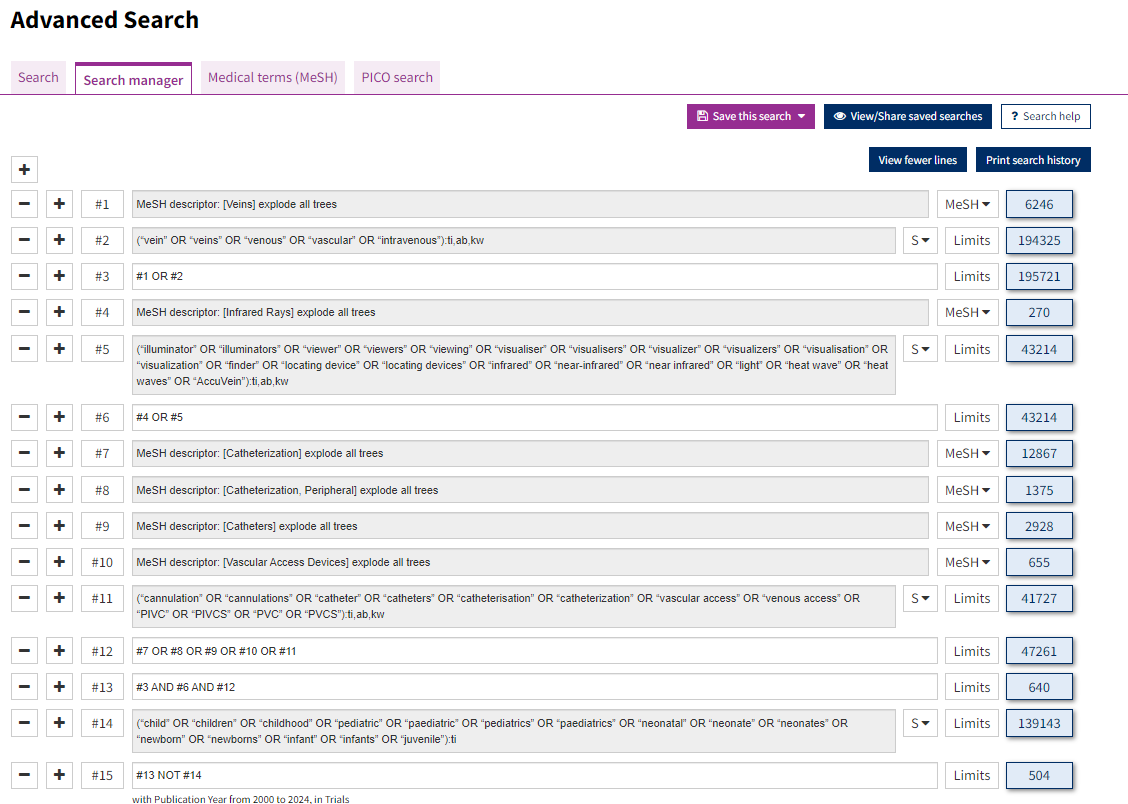


ID Search Hits

#1 MeSH descriptor: [Veins] explode all trees 6246

#2 (“vein” OR “veins” OR “venous” OR “vascular” OR “intravenous”):ti,ab,kw 194325

#3 #1 OR #2 195721

#4 MeSH descriptor: [Infrared Rays] explode all trees 270

#5 (“illuminator” OR “illuminators” OR “viewer” OR “viewers” OR “viewing” OR “visualiser” OR “visualisers” OR “visualizer” OR “visualizers” OR “visualisation” OR “visualization” OR “finder” OR “locating device” OR “locating devices” OR “infrared” OR “near-infrared” OR “near infrared” OR “light” OR “heat wave” OR “heat waves” OR “AccuVein”):ti,ab,kw 43214

#6 #4 OR #5 43214

#7 MeSH descriptor: [Catheterization] explode all trees 12867

#8 MeSH descriptor: [Catheterization, Peripheral] explode all trees 1375

#9 MeSH descriptor: [Catheters] explode all trees 2928

#10 MeSH descriptor: [Vascular Access Devices] explode all trees 655

#11 (“cannulation” OR “cannulations” OR “catheter” OR “catheters” OR “catheterisation” OR “catheterization” OR “vascular access” OR “venous access” OR “PIVC” OR “PIVCS” OR “PVC” OR “PVCS”):ti,ab,kw 41727

#12 #7 OR #8 OR #9 OR #10 OR #11 47261

#13 #3 AND #6 AND #12 640

#14 (“child” OR “children” OR “childhood” OR “pediatric” OR “paediatric” OR “pediatrics” OR “paediatrics” OR “neonatal” OR “neonate” OR “neonates” OR “newborn” OR “newborns” OR “infant” OR “infants” OR “juvenile”):ti 139143

#15 #13 NOT #14 with Publication Year from 2000 to 2024, in Trials 504

**CINAHL Complete (EBSCOhost)** 463 results

Includes CINAHL Subject Headings

((MH "Veins+" OR TI(“vein” OR “veins” OR “venous” OR “vascular” OR “intravenous”) OR AB(“vein” OR “veins” OR “venous” OR “vascular” OR “intravenous”)) AND (TI(“illuminator” OR “illuminators” OR “viewer” OR “viewers” OR “viewing” OR “visualiser” OR “visualisers” OR “visualizer” OR “visualizers” OR “visualisation” OR “visualization” OR “finder” OR “locating device” OR “locating devices” OR “infrared” OR “near-infrared” OR “near infrared” OR “light” OR “heat wave” OR “heat waves” OR “AccuVein”) OR AB(“illuminator” OR “illuminators” OR “viewer” OR “viewers” OR “viewing” OR “visualiser” OR “visualisers” OR “visualizer” OR “visualizers” OR “visualisation” OR “visualization” OR “finder” OR “locating device” OR “locating devices” OR “infrared” OR “near-infrared” OR “near infrared” OR “light” OR “heat wave” OR “heat waves” OR “AccuVein”)) AND (MH "Catheterization+" OR MH "Catheterization, Peripheral+" OR MH "Catheters+" OR MH "Vascular Access Devices+" OR TI(“cannulation” OR “cannulations” OR “catheter” OR “catheters” OR “catheterisation” OR “catheterization” OR “vascular access” OR “venous access” OR “PIVC” OR “PIVCS” OR “PVC” OR “PVCS”) OR AB(“cannulation” OR “cannulations” OR “catheter” OR “catheters” OR “catheterisation” OR “catheterization” OR “vascular access” OR “venous access” OR “PIVC” OR “PIVCS” OR “PVC” OR “PVCS”)) AND (LA (English OR French)) NOT ((MH "Animals+" OR MH "Animal Studies" OR TI animal model*) NOT MH "Human") AND PY 2000-2024) NOT (TI(“child” OR “children” OR “childhood” OR “pediatric” OR “paediatric” OR “pediatrics” OR “paediatrics” OR “neonatal” OR “neonate” OR “neonates” OR “newborn” OR “newborns” OR “infant” OR “infants” OR “juvenile”))

**Scopus (Elsevier)** 1629 results

Advanced document search

(TITLE-ABS (({vein} OR {veins} OR {venous} OR {vascular} OR {intravenous}) AND ({illuminator} OR {illuminators} OR {viewer} OR {viewers} OR {viewing} OR {visualiser} OR {visualisers} OR {visualizer} OR {visualizers} OR {visualisation} OR {visualization} OR {finder} OR {locating device} OR {locating devices} OR {infrared} OR {near-infrared} OR {near infrared} OR {light} OR {heat wave} OR {heat waves} OR {AccuVein}) AND ({cannulation} OR {cannulations} OR {catheter} OR {catheters} OR {catheterisation} OR {catheterization} OR {vascular access} OR {venous access} OR {PIVC} OR {PIVCS} OR {PVC} OR {PVCS})) AND NOT TITLE({child} OR {children} OR {childhood} OR {pediatric} OR {paediatric} OR {pediatrics} OR {paediatrics} OR {neonatal} OR {neonate} OR {neonates} OR {newborn} OR {newborns} OR {infant} OR {infants} OR {juvenile})) AND PUBYEAR AFT 1999 AND LANGUAGE(English OR French)
