# Supplementary material for: Infrared Devices Versus Traditional Palpation Approach for Peripheral Intravenous Catheter Insertion in Adults: A Systematic Review and Meta‐Analysis
Source: J Adv Nurs. 2025 Apr 29;82(2):1775–90. doi: 10.1111/jan.17007 (PMC12810616; doi:10.1111/jan.17007)
Supplement: Supplementary file 2 — File S2. Subgroup analysis for first attempt insertion success using infrared (IR) devices compared to traditional approach in (1) emergency departments (EDs) and (2) other wards. [file JAN-82-1775-s001.docx]

Supplementary file 2. Subgroup analysis for first attempt insertion success using infrared (IR) devices compared to traditional approach in (1) emergency departments (EDs) and (2) other wards.

(1)

(2)

ED: Emergency Department; IR: Infrared
