# Supplementary material for: Infrared Devices Versus Traditional Palpation Approach for Peripheral Intravenous Catheter Insertion in Adults: A Systematic Review and Meta‐Analysis
Source: J Adv Nurs. 2025 Apr 29;82(2):1775–90. doi: 10.1111/jan.17007 (PMC12810616; doi:10.1111/jan.17007)
Supplement: Supplementary file 3 — File S3. Sensitive analysis for first attempt insertion success using infrared (IR) devices compared to traditional approach: (1) without studies with high‐risk bias; (2) without the study with crossover design; (3) with fixed effect model analysis. [file JAN-82-1775-s003.docx]

Supplementary file 3. Sensitive analysis for first attempt insertion success using infrared (IR) devices compared to traditional approach: (1) without studies with high-risk bias; (2) without the study with crossover design; (3) with fixed effect model analysis.

(1)

(2)

(3)

IR: Infrared
